# Recurrent Soft Tissue Infections Associated With Burosumab Therapy in X-Linked Hypophosphatemic Rickets

**DOI:** 10.1210/jcemcr/luad120

**Published:** 2023-11-07

**Authors:** Sean Ho Yoon, Pasquale Passarella

**Affiliations:** Department of Endocrinology, Albany Medical Center, Albany, NY 12208, USA; Department of Endocrinology, Albany Medical Center, Albany, NY 12208, USA

**Keywords:** X-linked hypophosphatemic rickets, burosumab, fibroblast growth factor, soft tissue infection

## Abstract

X-linked hypophosphatemic rickets (XLH) is a genetic disorder characterized by elevated fibroblast growth factor 23 (FGF23), resulting in renal phosphate wasting and inadequate bone mineralization. Burosumab, a monoclonal antibody that inhibits FGF23 activity, has shown promise in improving renal phosphate reabsorption and clinical outcomes in XLH patients. However, the potential side effects of burosumab, particularly its impact on immune function and susceptibility to infections, remain a subject of concern. In this case report, we describe a 57-year-old male individual with XLH who experienced recurrent soft tissue infections while receiving burosumab therapy. The infections included an olecranon abscess, a cervical retropharyngeal phlegmon with a sternocleidomastoid abscess, and suprapubic cellulitis, all of which were treated with antibiotic therapy. Following discontinuation of burosumab therapy, the patient did not experience further soft tissue infections. These observations suggest a potential association between burosumab therapy and an increased risk of soft tissue infections. Mechanistically, disruption of the FGF23-Klotho signaling axis may lead to impaired humoral immunity mediated by B lymphocytes and compromised innate immune response mediated by macrophages. Further investigation is warranted to better understand the immunological effects of burosumab and its implications for infectious complications in XLH patients.

## Introduction

X-linked hypophosphatemic rickets (XLH) arises from a pathogenic variant of the PHEX gene, resulting in elevated fibroblast growth factor 23 (FGF23) levels due to impaired metabolism by endopeptidase. The increased FGF23 level leads to hypophosphatemia by promoting renal phosphate excretion and reducing vitamin D activation, subsequently hampering phosphate absorption [1]. Consequently, insufficient hydroxyapatite formation and inadequate bone mineralization occur, causing rickets in children and osteomalacia in adults. Patients with XLH exhibit various clinical manifestations, including short stature, deformities in weight-bearing limbs, arthralgia, and myalgia. Additionally, impaired dentin mineralization contributes to tooth abscesses and excessive dental caries [1].

Conventional treatment for XLH involves oral phosphate and active vitamin D analogues. However, oral phosphate replacement often necessitates multiple daily doses, potentially increasing the pill burden. Overcompensation with phosphate can result in secondary hyperparathyroidism, exacerbating existing metabolic bone disease, and can pose a risk for nephrocalcinosis when combined with high doses of active vitamin D analogues. Burosumab, a recombinant human IgG1 monoclonal antibody, binds to FGF23 and inhibits its activity. Studies have demonstrated that burosumab improves renal phosphate reabsorption, reduces the severity of rickets in children and osteomalacia in adults, and enhances the quality of life by alleviating myalgia and weakness [2]. Burosumab also has the potential to eliminate the need for multiple daily doses of phosphate and active vitamin D analogues, thereby reducing the pill burden and associated complications [2].

Clinical trials have identified certain side effects of burosumab, including local injection site reactions, hypersensitivity reactions such as rashes or urticaria, and restless leg syndrome [3]. Concerns have been raised regarding the altered immunity from burosumab due to its autoantibody action, but to date, no relevant adverse events have been observed or documented. Our case study highlights recurrent soft tissue infections during burosumab therapy, which may elucidate the first observed side effect associated with its use.

## Case Presentation

The patient is a 57-year-old man diagnosed with X-linked hypophosphatemic rickets (XLH) during his childhood through genetic and biochemical testing. He comes from a family with a strong history of the disease, including 3 other brothers who are affected. Initially, the patient did not seek medical attention, resulting in short stature, leg bowing, and the loss of multiple teeth during adolescence. Additionally, he developed degenerative arthritis in his lower extremities, as well as ossification of the ligamentum flavum in his thoracic vertebrae, leading to spinal stenosis that was treated with laminectomies. Ongoing muscle weakness, myalgia, and joint stiffness compelled the patient to rely on a walker or cane for mobility. Otherwise, the patient denied history of drug or alcohol abuse, recent hospitalizations, or human immunodeficiency virus (HIV) infection.

## Treatment

Considering the substantial impact of his symptoms on his quality of life, the patient initiated burosumab therapy at a dose of 1 mg/kg every 4 weeks. Serum phosphorus levels were monitored carefully and consistently remained within the normal range, except in the setting of brief dosage pausing during hospitalizations with the infection (Table 1). Renal ultrasound did not reveal any nephrolithiasis. Physical therapy sessions were continued to improve mobility.

**Table 1.** Serum adjusted calcium, serum phosphorus, and 25 hydroxy vitamin D over time

Dates collected	Serum adjusted calcium: 2.20-2.55 mmol/L (8.6-10.3 mg/dL)	Serum phosphorus: 0.77-1.52 mmol/L (2.4-4.7 mg/dL)	25 hydroxy vitamin D: 50-125 nmol/L (20-50 ng/mL)	Notes
June 21, 2019	2.30 mmol/L (9.2 mg/dL)	1.16 mmol/L (3.6 mg/dL)	78.25 nmol/L (31.3 ng/mL)	Burosumab started
July 26, 2019	2.30 mmol/L (9.2 mg/dL)	0.94 mmol/L (2.9 mg/dL)	—	
Jan. 13, 2020	2.27 mmol/L (9.1 mg/dL)	0.81 mmol/L (2.5 mg/dL)	79.75 nmol/L (31.9 ng/mL)	
Dec. 29, 2020	2.27 mmol/L (9.1 mg/dL)	—	62.25 nmol/L (24.9 ng/mL)	
Feb. 5, 2021	2.22 mmol/L (8.9 mg/dL)	0.71 mmol/L (2.2 mg/dL)	102.75 nmol/L (41.17 ng/mL)	
Oct. 10, 2021	2.35 mmol/L (9.4 mg/dL)	0.74 mmol/L (2.3 mg/dL)	—	Left olecranon abscess occurred
Jan. 24, 2022	2.27 mmol/L (9.1 mg/dL)	0.77 mmol/	—	Cervical abscess occurred
May 30, 2022	2.25 mmol/L (9.0 mg/dL)	0.87 mmol/L (2.7 mg/dL)	—	
Aug. 3, 2022	2.30 mmol/L (9.2 mg/dL)	0.81 mmol/L (2.5 mg/dL)	—	Suprapubic abscess occurred and burosumab discontinued
Oct. 4, 2022	2.22 mmol/L (8.9 mg/dL)	0.81 mmol/L (2.5 mg/dL)	—	
Jan. 24, 2023	2.25 mmol/L (9.0 mg/dL)	0.61 mmol/L (1.9 mg/dL)	96.75 nmol/L (38.7 ng/mL)	

## Outcomes and Follow-Up

**Figure 1.**
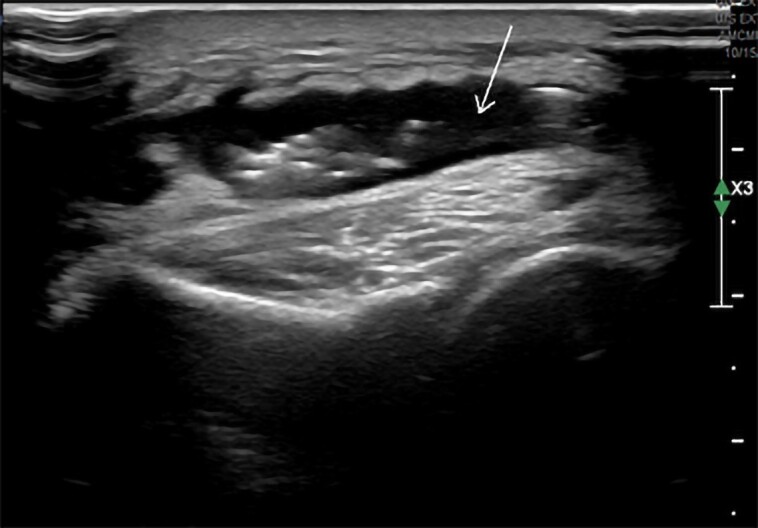
Ultrasound imaging of left olecranon abscess (arrow).

**Figure 2.**
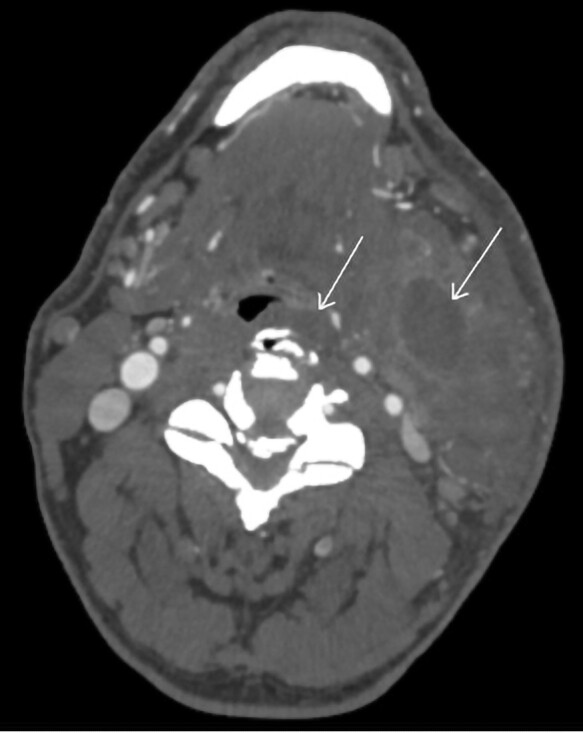
Computed tomography image of neck, demonstrating cervical retropharyngeal phlegmon (arrow on left) and abscess in the sternocleidomastoid muscle (arrow on right).

**Figure 3.**
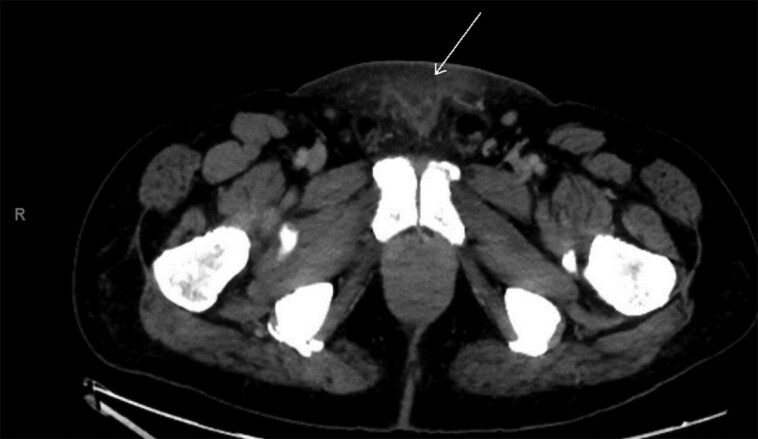
Computed tomography image of abdomen, demonstrating a marked fat stranding with skin induration of anterior suprapubic subcutaneous tissue compatible with cellulitis (arrow).

Approximately one year into the therapy, the patient developed a left olecranon abscess measuring 6.6 × 0.7 × 5.2 cm on ultrasound (Fig. 1). Subsequently, abscess drainage and left olecranon bursectomy were performed. Tissue culture confirmed the presence of methicillin-sensitive Staphylococcus aureus (MSSA), and the patient completed a short course of cephalexin. Three months later, the patient presented with dysphagia accompanied by swelling on the left side of his neck. A neck computed tomography (CT) scan revealed a cervical retropharyngeal phlegmon and a multiloculated abscess measuring 2.5 × 1.6 cm in the sternocleidomastoid muscle (Fig. 2). Emergency transcervical incision and drainage were carried out, resulting in the release of 20 cc of pus. Both blood and abscess drainage cultures grew methicillin-resistant Staphylococcus aureus (MRSA), and the patient received treatment with linezolid for several weeks. Upon cardiology evaluation, no cardiac murmurs were detected, and the electrocardiogram exhibited mild non-ST elevation, suggestive of transient demand ischemia. Considering the recurrent fevers linked to the MRSA infection, a transthoracic echocardiogram was conducted, which demonstrated the absence of valvular vegetations or regurgitation. After 6 months, the patient developed another erythematous and tender skin lesion in the suprapubic area. Abdominal and pelvic CT imaging revealed significant fat stranding with skin induration in the anterior subcutaneous tissue of the suprapubic area, suggestive of cellulitis (Fig. 3). No discrete, rim-enhancing, drainable abscess was found. Considering the patient's history of MRSA infection, empirical treatment with a 7-day course of linezolid was administered. Despite being on burosumab therapy, the patient reported no subjective improvement in his quality of life, lower extremity weakness, or myalgia. While it remained unclear whether burosumab was contributing to the recurrent soft tissue infections, due to its lack of evident clinical benefit, the therapy was discontinued. The patient was then switched to a daily dose of 0.5 mcg of calcitriol, accompanied by as-needed phosphate replacement, while continuing physical therapy. Following the discontinuation of burosumab, the patient did not experience any further recurrent soft tissue infections in subsequent years.

## Discussion

Clinical trials of burosumab involving pediatric and adult patients have described various side effects, some of which were local injection site reactions, hypersensitivity reactions including rashes or hives, tooth abscesses, and restless leg syndrome. However, soft tissue infection has not been observed or listed as an adverse effect in therapy labeling [1, 4]. Monoclonal antibodies (mAbs) that directly target antigens on B or T lymphocytes and tumor necrosis factor (TNF) are traditionally known to reduce immune function, potentially increasing the risk of various infections [5].

FGF23, synthesized from osteoblasts and osteocytes in the bone, interacts with the FGF receptor-Klotho protein complex, forming the FGF23-Klotho signaling axis that influences calcium-phosphorus metabolism. This signaling axis primarily affects the kidneys by promoting phosphaturia and inhibiting renal 1-α-hydroxylase, as well as the parathyroid glands by suppressing parathyroid hormone (PTH) secretion and synthesis [6].

Recent studies have revealed that the FGF23-Klotho signaling axis also influences immune function. A study conducted on mice lacking FGF23-Klotho signaling demonstrated a reduction in splenocyte numbers [7]. Flow cytometry and immunohistochemistry analyses identified cells expressing both FGF receptor and Klotho, including CD45R/B220+ CD21/CD35+ CD1d+ CD43− marginal zone B cells. This suggests that FGF23 binding may elicit a response in these Klotho-expressing B cells. In a mouse model, plasmacytoid dendritic cells in proximity to Klotho-positive B cells produced FGF23 [7]. These findings indicate that a recombinant human monoclonal antibody targeting FGF23, such as burosumab, may disrupt its immunological role in the spleen, specifically among B lymphocytes, leading to decreased humoral immunity against bacterial infections. Other studies have also suggested that FGF23 affects macrophages. Macrophages and dendritic cells express the FGF receptor, and inflammatory stimuli, such as bacterial infections, upregulate Klotho expression in these cells, establishing FGF23-Klotho signaling [8]. Downstream signaling induces TNF-α and innate immune responses by pro-inflammatory M1-activated macrophages, which are responsible for releasing inflammatory cytokines and directly acting against pathogens [9]. Therefore, inhibiting FGF23 could result in an ineffective macrophage-induced innate immune response against bacterial infections.

The recurrent bacterial soft tissue infections observed in this case during burosumab therapy can be explained by potential disruptions in both humoral immunity mediated by B lymphocytes in the spleen and innate immunity mediated by macrophages. In addition to its conventional role in calcium-phosphorus metabolism, FGF23 has been shown to have pleiotropic effects on the immune system. Drug-resistant bacterial infection, such as MRSA in this case, could be attributed to a healthcare-associated infection stemming from prior hospitalization. However, it is worth noting that episodes of multiple soft tissue infections are not typically observed among immunocompetent individuals. Overall, the presented case highlights the occurrence of recurrent soft tissue infections in a patient undergoing burosumab therapy for XLH. While burosumab has demonstrated effectiveness in improving renal phosphate reabsorption and quality of life in XLH patients, its potential adverse effects including the infection risk should be carefully monitored and considered. Future studies with larger sample sizes or more extended periods of burosumab therapy should be conducted to establish this adverse effect.

## Learning Points

- Burosumab therapy in XLH may be associated with an increased risk of recurrent soft tissue infections, which should be monitored closely in clinical practice.
- Disruption of the FGF23-Klotho signaling axis by burosumab may impact both humoral immunity mediated by B lymphocytes and innate immune response mediated by macrophages, potentially compromising the patient's ability to fight off bacterial infections.
- While burosumab has shown effectiveness in improving renal phosphate reabsorption and clinical outcomes in XLH patients, its potential adverse effects, such as an increased susceptibility to soft tissue infections, should be considered when evaluating the overall risk-benefit profile of the treatment. Further research is needed to elucidate the exact mechanisms behind the immunological effects of burosumab and the associated risk of infections.

## Contributors

All authors made individual contributions to authorship. S.Y. and P.P. were involved in the diagnosis and management of this patient and manuscript submission. All authors reviewed and approved the final draft.

## Data Availability

Original data generated and analyzed for this case report are included in this published article.

## Abbreviations

CT: computed tomography
FGF23: fibroblast growth factor 23
MRSA: methicillin-resistant Staphylococcus aureus
TNF: tumor necrosis factor
XLH: X-linked hypophosphatemic rickets
